# Optimizing Testing Time for Methaemoglobin Reduction Test in G6PD Screening: A Comparative Study of Monica Cheesbrough’s Protocol and a Modified Approach at a Hospital in a Resource‐Restraint Country, Ghana

**DOI:** 10.1155/ah/2919872

**Published:** 2025-11-20

**Authors:** Richard Vikpebah Duneeh, Emmanuel Appiah-Intsiful, Israel Bedzina, Elliot Elikplim Akorsu, Emmanuel Allotey, Mercy Adzo Klugah, Francis Agyei Amponsah, Wina Ivy Ofori Boadu, Paul Ntiamoah, Alexander Asamoah, Kenneth Ablordey

**Affiliations:** ^1^ Department of Medical Laboratory Sciences, School of Health and Allied Sciences, University of Health and Allied Sciences, Ho, Ghana, uhas.edu.gh; ^2^ Department of Medical Diagnostics, College of Health Sciences, Kwame Nkrumah University of Science and Technology, Kumasi, Ghana, knust.edu.gh; ^3^ Department of General and Liberal Studies, School of Basic and Biomedical Sciences, University of Health and Allied Sciences, Ho, Ghana, uhas.edu.gh; ^4^ Ghana Health Service, St. Elizabeth Catholic Hospital, Hwidiem, Ghana, ghanahealthservice.org; ^5^ Ghana Health Service, National Malaria Elimination Programme, Accra, Ghana, ghanahealthservice.org

**Keywords:** G6PD, intraclass correlation coefficient, methaemoglobin reduction test

## Abstract

**Background:**

Glucose‐6‐phosphate dehydrogenase (G6PD) deficiency is an inherited disorder caused by a genetic defect in the red blood cell enzyme G6PD, affecting around 500 million people worldwide. The study investigated the optimum Methaemoglobin Reduction Test (MRT) reaction time for diagnosing G6PD deficiency among patients at Asutifi North District Hospital using Monica Cheesbrough protocol and Asutifi North District Hospital Protocol.

**Methodology:**

The study was an experimental study conducted with 643 participants from April, 2024 to July, 2024. The Monica Cheesbrough MRT and the modified approach (Asutifi North Hospital MRT protocols), were compared at various time points (T90, T120, T150, and T180 min). Intraclass Correlation Coefficient (ICC) and Kappa statistics were used to assess reliability and agreement between the results from the two protocols. McNemar’s test was utilized to analyse G6PD status differences between the protocols. The prevalence of G6PD deficiency was also determined. Data analyses were done using IBM Statistical Package for the Social Sciences version 26.0 (2019. Armonk, NY: IBM Corp). *p*‐value less than 0.05 was considered statistically significant for all analyses.

**Results:**

T90 ICC values were very low (0.005–0.007), indicating poor agreement. From T120 onwards, ICC values were high (0.967–0.996, *p* < 0.001), demonstrating excellent reliability. Significant differences in G6PD status were found at T90 (*p* < 0.001), with diminishing differences at later time points. Kappa values indicated slight agreement at T90 (*K* = 0.164, *p* < 0.001) and perfect agreement from T120 onwards (*K* = 1.000, *p* < 0.001). The majority of participants 606 (94.2%) had normal G6PD status, 29 (4.5%) had a fully defective enzyme, and 8 (1.2%) had a partially defective enzyme activity.

**Conclusion:**

The study found the optimum MRT reaction to be 120 min. The study also emphasized lesser volumes of blood samples and reagent give accurate results in the optimum time established. These insights can contribute to faster turnaround times and efficient sample and reagent management especially amongst paediatric patients where it is difficult taking very large volumes of blood for testing.

## 1. Introduction

Glucose‐6‐phosphate dehydrogenase (G6PD) deficiency is a genetic disorder that results in an inadequate amount of G6PD enzyme in red blood cells (RBC), a biological catalyst that is important to produce the reduced form of Nicotinamide Adenine Dinucleotide Phosphate (NADP) that protects red blood cells against oxidative stress [1]. It is the commonest inherited enzymopathy that is particularly prevalent in malaria‐endemic regions [2]. A study in Mozambique; a malaria endemic region reported a considerably high (13.2%) G6PD deficiency prevalence [3].

G6PD gene is a partially dominant sex‐linked (X‐chromosome) gene, males show full enzyme deficiency expression, and are hemizygous while females usually exhibit intermediate expression and are heterozygous [4]. In females that are heterozygous for G6PD deficiency polymorphisms, the picture of cell population variation is more complicated due to the random X‐chromosome inactivation that produces a mosaic population of G6PD‐normal and G6PD‐deficient RBCs [5].

G6PD deficiency is particularly prevalent in Sub‐Saharan and Western Africa, with rates typically ranging from 10% to 20% or higher in these regions [6]. This high prevalence correlates with an exponential increase in anaemia cases, highlighting the significant health impact of this genetic condition. A recent study conducted in Ghana by Amoah et al. [7] exemplifies this trend, reporting a G6PD deficiency prevalence of 10.6% among children. These trends emphasize the importance of G6PD screening and management in African populations, especially given its association with increased risk of anaemia and other health complications [8].

Methaemoglobin is a form of haemoglobin that is unable to transport oxygen to the body tissues effectively, leading to tissue hypoxia and various health complications [9]. Methaemoglobin Reduction Test (MRT) has been widely used as the major screening test for G6PD deficiency in many parts of the world especially in resource restraint countries due to its low cost, simplicity and rapidity requirement [10]. It is particularly advantageous in resource‐restraint settings due to its cost‐effectiveness and simplicity [10]. Studies have shown that the MRT can reliably identify G6PD deficient individuals when compared with more complex methods like spectrophotometry [10].

The test measures the time taken for methylene blue to reduce methaemoglobin to haemoglobin [11]. Methylene blue activates the pentose phosphate pathway (PPP), resulting in the enzymatic conversion of methaemoglobin back to haemoglobin in those red cells with normal G6PD activity [10].

At the Asutifi North District Hospital and most laboratories in Ghana, there is a significant gap with regards to the volume of blood and the required time for MRT using Monica Cheesbrough protocol. Therefore, this study investigated the appropriate test time, blood volume, and reagent volume required for the MRT at the Asutifi North District Hospital, in order to address the lack of standardization and improve the reliability of the MRT.

## 2. Methodology

### 2.1. Study Design

This study utilized an experimental cross‐sectional design from April 2024 to July 2024 to determine the optimum MRT time for G6PD deficiency screening. The study compared the Monica Cheesbrough MRT and the modified approach (Asutifi North Hospital MRT) protocols using different blood and reagent volumes.

### 2.2. Study Site

This study was conducted at the Asutifi North District Hospital in the Kenyasi District of the Ahafo Region, Ghana, which serves a population of about 52,259 people. The Asutifi North District itself lies between latitudes 6°40′–7°15′ North and longitudes 2°15′–2°45′ West, covering a land area of 936 sq.km, and shares boundaries with several districts and municipalities, making it one of the smallest in the former Brong Ahafo Region. [12]. The hospital has key departments, including medical, surgical, paediatric, and maternity wards, as well as laboratory services, which were directly involved in this study.

### 2.3. Study Population

The study population for this study were individuals who were being screened for G6PD deficiency. This included newborns, children, pregnant women and adults seeking care at the Asutifi North District Hospital during the study period.

### 2.4. Inclusion Criteria

This study included newborns, children and adults who sought care at the Asutifi North District, signed consent and child assent to participate.

### 2.5. Exclusion Criteria

All individuals who were transfused of blood 6 months prior to the test were excluded. Individuals who were severely ill were also excluded. Individuals who refused consent were also excluded.

### 2.6. Sample Size

Using the Raosoft online sample size calculator (https://www.raosoft.com/samplesize.html) with a population of 52,259 [12], 95% confidence interval, response distribution of 50% and a 5% error margin, a sample size of 382 was obtained. A total of 643 study participants were recruited to improve the power of the analysis.

### 2.7. Sampling Technique

The study utilized a convenient sampling protocol to select the 643 participants. This technique allowed for easy accessibility to individuals who were readily available at the study site, making recruitment faster and more practical within the study period.

### 2.8. Data and Sample Collection

Data collection involved obtaining written informed consent from adults and assent from minors, after which demographic information (age, sex) was recorded on data collection sheet and blood samples collected for G6PD testing using both Monica Cheesbrough MRT and the Asutifi North Hospital MRT protocols, with results read at 90, 120, 150, and 180 min. For blood collection, 9 mL of venous blood was drawn aseptically into three EDTA anticoagulated tubes using the vacutainer method of blood collection following standard protocols to ensure safety and accuracy. The procedure included vein selection, site disinfection, application of a tourniquet, insertion of a vacutainer needle, and careful mixing of the blood with anticoagulant. Samples were properly labelled with study codes, date, and time, then transported to the haematology unit of Asutifi North District Hospital for G6PD analysis, with strict adherence to protocols minimizing complications and ensuring participant comfort.

### 2.9. Principle of G6PD Test

Haemoglobin is oxidized to methaemoglobin (Hi) by sodium nitrite. The redox dye, methylene blue activates the pentose phosphate pathway, resulting in the enzymatic conversion of Hi back to haemoglobin in those red cells with normal G6PD activity. In G6PD deficient cells there is no enzymatic reconversion of Hi to haemoglobin [11].

### 2.10. Monica Cheesbrough’s G6PD Test Procedure and Modified Approach (Asutifi North District Hospital G6PD Test Procedure)

The G6PD test procedure, by Monica Cheesbrough and modified at Asutifi North District Hospital, involves the use of three glass tubes labelled Test, Normal, and Deficient, each prepared with varying amounts of sodium nitrite‐glucose reagent, methylene blue reagent, and patient’s blood. In Cheesbrough’s method, 0.1 mL reagents and 2 mL blood were used for each of the glass tubes [11], while the modified approach halved the volumes to 0.05 mL reagents and 1 mL blood for each of the glass tubes. After gentle mixing, the tubes were incubated at 37°C for 90 min, followed by transferring small aliquots from each tube into larger tubes containing distilled water (10 mL in Cheesbrough’s method and 5 mL in the modified approach). The colour changes in the solutions were then assessed at intervals (T90, T120, T150, and T180): similarity to the red colour of the Normal tube indicated normal G6PD activity, similarity to the brown Deficient tube indicated deficient or reduced activity, and intermediate colours suggested partial activity.

### 2.11. Data Handling and Analysis

Participant demographics and laboratory analysis results were entered, cleaned and coded using Microsoft office version 2021 and kept on a password‐protected computer accessible to the researchers only. The Excel file was exported into IBM Statistical Package for the Social Sciences version 26.0 (2019. Armonk, NY: IBM Corp) for statistical analysis. Categorical variables were presented as frequencies and percentages; a bar chart was used to present the prevalence of G6PD deficiency among participants. Intraclass correlation coefficient was used to assess the reliability of results between time intervals in each protocol. Kappa test was done to assess the reliability of differences in the results of the two protocols. Mcnemar *t* test was also performed to assess the difference in results between the two protocols across time intervals (T90, T120, T150, T180). A *p*‐value < 0.05 was considered statistically significant for all analyses.

### 2.12. Ethical Consideration

Ethical approval was obtained from the Research Ethics Committee (REC) of University of Health and Allied Sciences (UHAS) with reference number UHAS‐REC A.7 [18] 23‐24. Data was collected from participants who signed informed consent to participate. Confidentiality of the participants information was maintained by assigning special study numbers. In addition, the participants were made to understand that they have the right to withdraw from this study at any point should they feel distressed or threaten and suffer no consequences. Participation in the study was voluntary. The Helsinki declaration was observed as required.

## 3. Results

### 3.1. Sociodemographic Characteristics of Study Participants

Table 1 below shows the sociodemographic characteristics of 643 study participants. The median age of the participants was 25 years, with a wide range from 7 to 59 years. The age distribution showed that the largest Group 273 (42.5%) was between 21 and 30 years old, followed by 11–20 years 175 (27.2%), and 31–40 years 141 (21.9%). Smaller proportions were seen in the ≤ 10 years 17 (2.6%) and ≥ 40 years 37 (5.8%) age groups. There was a significant gender imbalance in the study population, with females comprising the vast majority at 569 (88.5%), while males made up only 74 (11.5%).

**Table 1:** Sociodemographic characteristics of study participants.

Variables	Frequency	Percentage (%)
Total	643	100.0
Age (years)		
Median (minimum‐maximum)	25 (7–59)	
≤ 10	17	2.6
11–20	175	27.2
21–30	273	42.5
31–40	141	21.9
≥ 40	37	5.8
Sex		
Female	569	88.5
Male	74	11.5

### 3.2. Intraclass Correlation Coefficient of G6PD in Both Protocols Over Time

Comparisons between T120, T150, and T180 for both protocols showed extremely high ICC values (ranging from 0.967 to 0.996), all with highly significant *p*‐values (*p* < 0.001) (Table 2).

**Table 2:** Intraclass correlation coefficient of G6PD in both protocols over time.

Variables	ICC [95% CI]	*p*‐value	Cronbach’s alpha
Monica cheesbrough MRT (minutes)			
T90 vrs. T120	0.007 [−0.159–0.150]	0.462	0.007
T90 vrs. T150	0.007 [−0.159–0.150]	0.463	0.007
T90 vrs. T180	0.007 [−0.159–0.150]	0.464	0.007
T120 vrs. T150	0.971 [0.967–0.976]	**< 0.001**	0.971
T120 vrs. T180	0.967 [0.962–0.972]	**< 0.001**	0.967
T150 vrs. T180	0.996 [0.995–0.996]	**< 0.001**	0.996
Asutifi north hospital MRT (minutes)			
T90 vrs. T120	0.005 [−0.161–0.148]	0.473	0.005
T90 vrs. T150	0.005 [−0.162–0.148]	0.475	0.005
T90 vrs. T180	0.005 [−0.162–0.148]	0.476	0.005
T120 vrs. T150	0.985 [0.983–0.987]	**< 0.001**	0.985
T120 vrs. T180	0.976 [0.973–0.980]	**< 0.001**	0.976
T150 vrs. T180	0.991 [0.989–0.992]	**< 0.001**	0.991

*Note:* Vrs; Versus, T; Time, *p* value is statistically significant at *p* < 0.050. Bold values are values that are statistically significant.

Abbreviations: ICC = Intraclass Correlation Coefficient, MRT = Methaemoglobin Reductase Test.

### 3.3. Differences in G6PD Status Between the Two Protocols Using McNemar *t*‐Test Over Time

Significant differences (*p* < 0.001) were observed at 90 min for all comparisons. At 120 min, a significant difference was found only in the No Defect (ND) category (*p* < 0.001), while Full Defect (FD) categories showed no significant differences. For the 150‐ and 180 min time points, significant differences were noted in the ND category (*p* < 0.001) when compared to the 90 min results of the Asutifi North Hospital test, but no significant differences were observed in other comparisons. The most pronounced differences occurred at the 90 min mark, with fewer divergences noted at later time points (Table 3).

**Table 3:** Differences in G6PD status between the two protocols using McNemar *t*‐test over time.

Asutifi north hospital methaemoglobin reduction test												
Monica cheesbrough methaemoglobin reduction test	90 min			120 min			150 min			180 min		
	ND *n* (%)	FD *n* (%)	*p*‐value	ND *n* (%)	FD *n* (%)	*p*‐value	ND *n* (%)	FD *n* (%)	*p*‐value	ND *n* (%)	FD *n* (%)	*p*‐value
90 min												
ND	1 (9.1)	10 (90.9)	**0.002**	11 (100.0)	0 (0.0)	**< 0.001**	11 (100.0)	0 (0.0)	**< 0.001**	11 (100.0)	0 (0.0)	**< 0.001**
FD	0 (0.0)	632 (100.0)		593 (85.7)	39 (5.6)		953 (96.1)	37 (3.7)		595 (94.1)	37 (5.9)	
120 min												
ND	1 (0.2)	604 (99.8)	**< 0.001**	604 (99.8)	1 (0.2)	1.000	605 (100.0)	0 (0.0)	1.000	605 (100.0)	0 (0.0)	1.000
FD	0 (0.0)	38 (100.0)		0 (0.0)	38 (100.0)		1 (2.6)	37 (97.4)		1 (2.6)	37 (97.4)	
150 min												
ND	1 (0.2)	605 (99.8)	**< 0.001**	604 (99.7)	2 (0.3)	0.500	606 (100.0)	0 (0.0)	1.000	606 (100.0)	0 (0.0)	1.000
FD	0 (0.0)	37 (100.0)		0 (0.0)	37 (100.0)		0 (0.0)	37 (100.0)		0 (0.0)	37 (100.0)	
180 min												
ND	1 (0.2)	605 (99.8)	**< 0.001**	604 (99.7)	2 (0.3)	0.500	606 (100.0)	0 (0.0)	1.000	606 (100.0)	0 (0.0)	1.000
FD	0 (0.0)	37 (100.0)		0 (0.0)	37 (100.0)		0 (0.0)	37 (100.0)		0 (0.0)	37 (100.0)	

*Note:* Partial defect and full defect = FD, *p* value is statistically significant at *p* < 0.050. Bold values are values that are statistically significant.

Abbreviations: FD = Full Defect, ND = No Defect.

### 3.4. Measurement of Agreement Between the Two Protocols Over Time

The agreement was measured using Kappa (K) statistics at 90, 120, 150, and 180 min. The results showed varying levels of agreement across different time points. At 90 min, there was slight agreement (*K* = 0.164) between the two protocols, which was statistically significant (*p* < 0.001). However, at later time points (120, 150, and 180 min), the agreement became much stronger, with Kappa values reaching as high as 1.000, indicating perfect agreement. These higher agreements were also statistically significant (*p* < 0.001) (Table 4).

**Table 4:** Measurement of agreement between the two protocols over time.

Asutifi north hospital methaemoglobin reduction test								
Monica cheesbrough MRT protocol	90 min		120 min		150 min		180 min	
	Kappa (K)	*p*‐value	Kappa (K)	*p*‐value	Kappa (K)	*p*‐value	Kappa (K)	*p*‐value
90 min	0.164	**< 0.001**	0.002	0.850	0.002	0.408	0.002	0.408
120 min	0.000	0.802	0.986	**< 0.001**	0.986	**< 0.001**	0.986	**< 0.001**
150 min	0.000	0.805	0.972	**< 0.001**	1.000	**< 0.001**	1.000	**< 0.001**
180 min	0.000	0.805	0.972	**< 0.001**	1.000	**< 0.001**	1.000	**< 0.001**

*Note:* *p* value is statistically significant at *p* < 0.050. Bold values are values that are statistically significant.

### 3.5. Prevalence of G6PD Deficiency Among Participants

**Figure 1:**
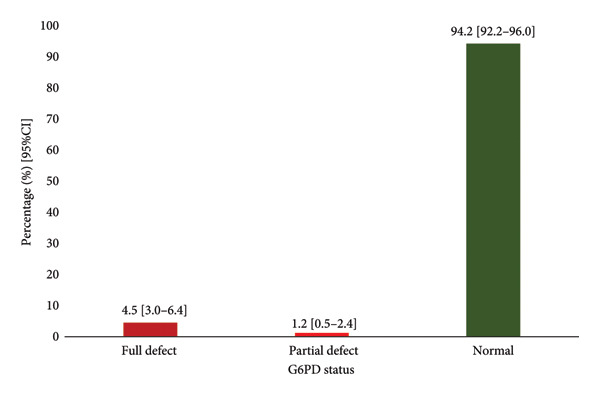
Prevalence of G6PD deficiency among participants. G6PD; glucose 6 phosphate dehydrogenase, prevalence was obtained from the 180^th^ min from both protocols (*K* = 1.000, *p* < 0.001).

Majority of the participants had normal G6PD enzyme 94.2 (95% CI; 92.2–96.0) whiles 4.5% (95% CI; 3.0–6.4) had a fully defective G6PD enzyme and 1.2% (95% CI; 0.5–2.4) had partially defective G6PD enzyme (Figure 1).

### 3.6. Distribution of G6PD Status Among Study Participants

Out of 643 total participants, the majority 606 (94.2%) had normal G6PD status, while 8 (1.2%) had a partial defect and 29 (4.5%) had a full defect. The age distribution showed that G6PD deficiency was not observed in participants aged 10 or younger, while it was most prevalent in the 21–30 age group and those 40 or older. Interestingly, all male participants 74 (100.0) had normal G6PD status, whereas females were partially defective and fully defective (1.2%, 4.5%) (Table 5).

**Table 5:** Distribution of G6PD status among study participants.

Variables	Normal *n* (%)	Partial defect *n* (%)	Full defect *n* (%)	*p*‐value
Total	606 (94.2)	8 (1.2)	29 (4.5)	
Age (years)				
≤ 10	17 (100.0)	0 (0.0)	0 (0.0)	
11–20	167 (95.4)	3 (1.7)	5 (2.9)	
21–30	254 (93.0)	5 (1.8)	14 (5.1)	0.554
31–40	134 (95.0)	0 (0.0)	7 (5.0)	
≥ 40	34 (92.0)	0 (0.0)	3 (8.0)	
Sex				
Female	532 (93.5)	8 (1.4)	29 (5.1)	
Male	74 (100.0)	0 (0.0)	0 (0.0)	0.788

*Note:* *p*‐value is significant at *p* > 0.050.

## 4. Discussion

The study aimed to investigate the optimum MRT reaction time and the corresponding volume of blood for diagnosing G6PD deficiency among patients attending the Asutifi North District Hospital in Ghana. The findings from the study are significant and provide valuable insights into the reliability and consistency of G6PD testing with the two protocols, the prevalence of G6PD deficiency, and the optimum time for accurate testing.

The analysis of the G6PD status at different time revealed that the measurements at 90 min were unreliable. However, the ICC values for T120, T150, and T180 were extremely high with highly significant *p*‐values (*p* < 0.001). This demonstrates that G6PD measurements become highly consistent and reliable after 120 min for both the Monica Cheesbrough MRT and the Asutifi North Hospital MRT. This consistency is crucial for accurate diagnosis and highlights that measurements taken at 90 min are not suitable for clinical decision‐making.

The study showed significant differences in G6PD status between the two protocols, especially at the 90‐min mark, where McNemar’s test indicated significant differences (*p* < 0.001) for all comparisons. At 120 min, significant differences were found only in the No Defect (ND) category. The agreement improved significantly at 150 and 180 min, with no significant differences observed in comparisons other than with the 90‐min results of the Asutifi North Hospital test. This suggests that while both protocols may yield different results in the early stages, their alignment improves significantly over time, making later time points more reliable for diagnosis. A strong agreement at 120 min and beyond with kappa statistics underscores the importance of allowing sufficient reaction time for the tests to produce consistent and reliable results.

The prevalence of G6PD deficiency among the participants was relatively low, with 4.5% having a fully defective enzyme, and 1.2% having a partially defective enzyme. These findings indicate that G6PD deficiency is not highly prevalent in the study population, but the presence of fully and partially defective cases underscores the need for accurate and reliable testing protocols to identify and manage affected individuals.

The prevalence of G6PD deficiency varies among different populations. Other studies in Ghana have reported G6PD deficiency prevalences ranging from about 10.6% [7] among school children in the south to 19.3% among pregnant women (2.3% full defect, 17.0% partial defect) [13]. Another study conducted among Cameroonian blood donors also found G6PD deficiency to be 7.9% [14].

In Kerman City, southern Iran, the prevalence was found to be 6.7%, with a higher incidence in males compared to females [15]. Among African American paediatric patients, the prevalence was 9% [4]. In Korea, the prevalence of decreased G6PD activity was 0.4% [16]. In south Gujarat, the incidence of G6PD deficiency was 3.69%, with a higher percentage in males compared to females [17]. Additionally, in the regions of Hefei, Fuyang, and Anqing in China, the overall prevalence rate was 0.09%, with varying rates among the different cities [18].

The study’s results clearly indicate that the optimum testing time for accurate G6PD status determination is 120 min. Measurements taken at or beyond this time point showed excellent reproducibility and consistency across both testing protocols. The agreement between the two protocols at 150 and 180 min was particularly strong, suggesting that extending the testing time beyond 120 min can further enhance diagnostic accuracy.

## 5. Conclusion

In conclusion, the study provides robust evidence that the optimum reaction time for the MRT with the two protocols for G6PD screening is 120 min. The findings emphasize the need for sufficient testing duration to ensure diagnostic accuracy and reliability. Lesser volumes of blood samples (1 mL in each tube and lower volumes of reagent 0.05 mL) give accurate results in the optimum time established. These insights can contribute in faster turnaround times and efficient sample and reagent management especially amongst paediatric patients where it is difficult taking very large volumes of blood for testing.

### 5.1. Limitations

Despite the robust findings, this study has some limitations. The study was conducted in a single hospital setting, which may limit the generalizability of the results to other healthcare facilities with different patient demographics or testing protocols.

### 5.2. Recommendations

Based on these limitations, future studies should aim to include diverse populations across multiple healthcare settings to enhance the generalizability of the findings. It is also recommended to explore additional statistical protocols to complement ICC and Kappa statistics, providing a more comprehensive understanding of the agreement between testing protocols. Researchers should consider controlling for or systematically documenting external factors that could impact test results. Additionally, developing standardized protocols and training for professionals performing G6PD tests can help minimize variability and improve the consistency.

**Nomenclature**
G6PD: Glucose‐6‐phosphate dehydrogenase
NADP: Nicotinamide adenine dinucleotide phosphate
RBC: Red blood cells
ICC: Intraclass correlation coefficient
MRT: Methaemoglobin reduction test
ICU: Intensive care units
NICU: Neonatal intensive care units

## Consent

The authors have nothing to report.

## Disclosure

A preprint has previously been published [19].

## Conflicts of Interest

The authors declare no conflicts of interest.

## Author Contributions

Conceptualization: Richard Vikpebah Duneeh.

Methodology: Kenneth Ablordey, Emmanuel Appiah‐Intsiful, Israel Bedzina, Paul Ntiamoah, and Mercy Adzo Klugah.

Formal analysis: Kenneth Ablordey.

Resources: Kenneth Ablordey, Richard Vikpebah Duneeh, Israel Bedzina, Wina Ivy Ofori Boadu, Francis Agyei Amponsah, Emmanuel Allotey, Mercy Adzo Klugah, and Elliot Elikplim Akorsu.

Project administration: Richard Vikpebah Duneeh, Francis Agyei Amponsah, Israel Bedzina, and Paul Ntiamoah.

Investigation: Emmanuel Appiah‐Intsiful, Kenneth Ablordey, Wina Ivy Ofori Boadu, Alexander Asamoah, and Paul Ntiamoah.

Writing–original draft: Kenneth Ablordey, Francis Agyei Amponsah, and Israel Bedzina.

Writing–review and editing and supervision: Richard Vikpebah Duneeh, Mercy Adzo Klugah, Francis Agyei Amponsah, Wina Ivy Ofori Boadu, Emmanuel Allotey, and Elliot Elikplim Akorsu.

## Funding

The authors received no external funding for this work.

## Data Availability

All relevant data will be made available upon request from corresponding author.
